# Co-Inoculation of *Mesorhizobium ciceri* with Either *Bacillus* sp. or *Enterobacter aerogenes* on Chickpea Improves Growth and Productivity in Phosphate-Deficient Soils in Dry Areas of a Mediterranean Region

**DOI:** 10.3390/plants10030571

**Published:** 2021-03-17

**Authors:** Imane Benjelloun, Imane Thami Alami, Mohamed El Khadir, Allal Douira, Sripada M. Udupa

**Affiliations:** 1 Department of Microbiology, National Institute of Agronomical Research (INRA), 10 000 Rabat, Morocco; i.benjelloun@hotmail.fr (I.B.); imane.thamialami@inra.ma (I.T.A.); mohamed_elkhadir@caramail.com (M.E.K.); 2 Department of Biology, Faculty of Sciences, Ibn Tofail University, 14 020 Kénitra, Morocco; douiraallal@hotmail.com; 3 ICARDA-INRA Cooperative Research Project, International Center for Agricultural Research in the Dry Areas (ICARDA), 10 000 Rabat, Morocco

**Keywords:** plant–microbe interactions, plant growth-promoting rhizobacteria, beneficial microbes, chickpea, bio-fertilizers, nitrogen fixation, phosphate solubilization, phosphate deficiency

## Abstract

Biological nitrogen fixation requires a large amount of phosphorus (P). However, most of the soils are P-deficient and the extensive use of P- chemical fertilizers constitute a serious threat to the environment. In this context, two field experiments were carried out to investigate the effect of co-inoculation of *Mesorhizobium ciceri* with phosphate solubilizing bacteria (PSB), *Bacillus* sp., and *Enterobacter aerogenes,* on chickpea as an alternative to chemical nitrogen (N) and phosphorous fertilizers in P-deficient soils in dry areas of Morocco. The results revealed that combined inoculation of chickpea with rhizobia and PSB showed a significant enhancement of chickpea nodulation, biomass production, yields and N, P, and protein content in grains as compared to single inoculation or single application of N or P. A significantly higher increase was obtained by inoculating chickpea with *Mesorhizobium* sp. MA72 combined with *E. aerogenes* P1S6. This combination allowed an enhancement of more than 270% in nodulation, 192% in shoot dry weight and 242% in grain yield. The effect of this combination was equivalent to the effect of combined application of N and P fertilizers. Formulation of biofertilizers based on tasted strains could be used for chickpea co-inoculation in P-deficient soils for an eco-friendly sustainable production of chickpea.

## 1. Introduction

Chickpea (*Cicer arietinum* L.), also called Bengal gram or garbanzo bean, is the third most produced food legume in the word, and covers an area of approximately 17.8 Million ha in 46 countries [1]. Chickpea production is predominant in semi-arid regions across the Indian subcontinent, Pakistan, Turkey, North Africa, Mexico, Middle East, Southern Europe, Canada, USA, and Australia. Morocco produced 36.3 × 10^3^ tons of chickpea in a producing area of 86.8 × 10^3^ ha during 2018 [1]. It is an ancient crop that holds an important place in the Mediterranean diet [2].

Chickpea is reported to have several potential human nutritional and health benefits, particularly for developing countries. It is considered as a cheap source of high-quality proteins, minerals (potassium, zinc, iron, calcium, magnesium, and phosphorus), carbohydrates, vitamins, and unsaturated fatty acids (linoleic acid and oleic acid) [3,4].

In addition to its nutritional quality, chickpea plays a major role in sustainable eco-friendly agriculture, because of its ability to fix atmospheric nitrogen through symbiotic associations with rhizobia. Chickpea can fix 20–80% of its nitrogen requirement [5,6], which enhances its productivity and improves soil N content [6,7,8]. However, the establishment of this symbiosis is limited by environmental conditions such as drought, salinity, extreme temperatures, soil acidity [9,10,11,12] and nutrient availability such as nitrogen (N) and phosphate (P) [13,14]. These abiotic stresses may cause damage to the host plant and reduce rhizobial survival [15,16], especially that chickpea in Mediterranean regions is grown as rainfed crop in semi-arid areas prone to water stress and exposed to increasingly variable and extreme weather conditions [17].

To alleviate the effect of these stresses, it is necessary to combine stress tolerant cultivars and stress tolerant rhizobacteria [9]. Moreover, the co-inoculation of rhizobia with other plant growth promoting rhizobacteria (PGPR) such as *Pseudomonas, Azotobacter, Bacillus, Erwinia, Serratia*, etc. can be used as an alternative to decrease abiotic stresses in legumes [18,19,20].

PGPRs might be found either in the rhizosphere, in the phyllosphere or inside nodules in the endosphere [9,21,22,23,24]. They mediate plant growth promotion directly through phosphate solubilization, nitrogen fixation, and phytohormones synthesis [14,19,25], or indirectly by acting as biocontrol agents either by competing for niches and nutrients or by producing antimicrobial metabolites, siderophores, etc. [23,26]. Under stress conditions, PGPRs act also through the production of deaminase 1-aminoacylclopropane-1-carboxylate (ACC) that regulate ethylene concentration and thus prevent its harmful accumulation in plant tissue and its negative effect on plant growth and development [20,27]. They were also reported to be involved in bioremediation of polluted soils through sequestering heavy metals and degrading xenobiotic compounds [28,29].

To take full advantage of nodulation and nitrogen fixation, legumes need phosphorus since these processes are very energy requiring, demanding at least 16 molecules of ATP for each molecule of N_2_ reduction [14,30,31]. Phosphorus (P) is also required for root development, nutrient uptake, legumes growth, development, and grains production [32,33,34]. Phosphorus was also reported to be involved in many metabolic processes such as photosynthesis [35,36]. However, most of the agricultural soils are P-deficient, which affects legumes production [37], causing major limits for nodule formation, biological nitrogen fixation (BNF), plant growth and productivity [13,38]. In P-deficient soils, the available amount of P is not enough to carry out efficient biological fixation. Rotaru and Sinclair [39] reported that symbiotic plants required more P than non-symbiotic plants because of the crucial role of P in nodules energetic transformations. Furthermore, many studies reported a significant correlation between P concentration in nodules and biological nitrogen fixation [39,40]. In addition, Meena et al. [41] reported that, under P limiting conditions, allocation of available P in nodules is more important for optimal symbiotic interaction. Thereby, the supply of available P is crucial for legumes-rhizobial associations.

The plant nutrition on P-deficient soils is usually achieved by the use of chemical fertilizers. Nevertheless, the high cost of these fertilizers and their negative environmental impacts are the two major constraints for their application [42,43]. Moreover, the excessive application of P fertilizers results in soil degradation by disturbing soil microbial diversity [44]. Furthermore, plants absorb less applied P fertilizer, and the rest is converted into insoluble P in the soil [28]. Phosphate solubilizing bacteria (PSB) have been reported to play a crucial role in increasing P availability in P-deficient soils by solubilizing the fixed P and supply it to plants [14,31,45]. *Bacillus, Enterobacter, Burkholderia, Erwinia, Pseudomonas, Serratia, and Rhizobium* were reported to be the genera with the most solubilizing P bacteria [46]. The use of bioinoculum based on competitive and efficient rhizobia fixing atmospheric nitrogen combined with P solubilizing bacteria may offer a viable promiscuous and advantageous alternative to N and P chemical fertilizers for a sustainable eco-friendly agriculture. 

The effect of co-inoculation of legumes with rhizobia and phosphate solubilizing bacteria has been largely reviewed by various authors [23,28,47,48,49,50,51]. Several workers reported that this co-inoculation stimulate plant growth and nodulation more than separate inoculation [48,52,53,54,55]. Zaheer et al. [22] reported that co-inoculation of chickpea with *Serratia* enhanced successfully plant growth and nodulation in a nutrient deficient soil. Similarly, Korir et al. [48] reported that co-inoculation of rhizobium strains with *Paenibacillus polymyxa* and *Bacillus megaterium* enhanced the growth of common bean in a phosphorous deficient soil. Furthermore, Raklami et al. [50] reported the positive effect of co-inoculation with rhizobia and PGPRs in mediterranean semi-arid regions of Morocco on biomass, growth, nodulation, and yield of faba been. Likewise, Sibponkrung et al. [56] reported that co-inoculation of *Bacillus velezensis* S141 with *Bradyrhizobium* USDA110 into soybean resulted in enhanced nodulation and N2-fixing efficiency. Singh et al. [57] reported the positive effect of a mixed inoculation of *M. ciceri* with *E. aerogenes* and *P. cypripedii,* on the growth and seed number of chickpea under pots experiment.

Most of the above and other studies on co-inoculation of chickpea with rhizobia and PGPRs were conducted under pot culture experiments and not many studies were reported under natural field conditions in P deficit soils of dry areas of Mediterranean regions. Here, we report the effect of inoculation of *Mesorhizobium* spp. with *Bacillus* sp. and for the first time with *Enterobacter aerogenes* on chickpea nodulation, biomass production, growth, grain yield, and nutrient uptake in P-deficient soils, under field conditions at two different locations. 

In this study, we hypothesized that combined inoculation of nitrogen fixing bacteria *Mesorhizobium* spp. and bacteria solubilizing phosphate would be able to increase growth, nodulation parameters and yield of chickpea more efficiently than separate single inoculation or P and N application.

## 2. Results

### 2.1. Effect of Co-Inoculation and Application of N and P Fertilization of Chickpea on Nodulation, Percent of Nitrogen Derived from Air, Shoot, and Root Dry Weight under Low Phosphate Conditions

**Figure 1:**
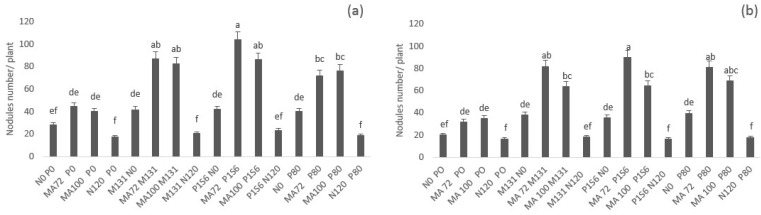
Effect of co-inoculation with *Mesorhizobium* spp. (MA72 or MA100) and *Bacillus* sp. (M131) or *E. aerogenes* (P1S6) and phosphate application combined to rhizobial inoculation on the number of nodules of chickpea in Merchouch (**a**) and Ain Sbit (**b**) sites. Bars followed by the same letter are not significantly different at α = 0.05. (P80 indicate application of 80 kg P2O5/ha, N120 indicate application of 120 kg N /ha, N0 and P0 indicate no nitrogen and phosphate fertilizers application).

The results showed that in both sites chickpea inoculation with rhizobia MA72 and MA100 enhanced nodule number and dry weight compared to non-inoculated and non-fertilized control. However, this single inoculation with rhizobia alone does not induce a clear increase of nodulation except for treatments fertilized with phosphate. These treatments showed an improvement of nodulation as a result of phosphate application (Figure 1).

Dual inoculation with rhizobia (MA72 or MA100) and PSB (M131 or P1S6) resulted in greater nodule number and dry weight compared to control and to single inoculation with rhizobia (Figure 1, Tables S1 and S2). Co-inoculation with MA72 and P1S6 resulted in extra abundant nodulation of chickpea; this combination enhanced nodulation by 271% and 305% in Merchouch and Ain Sbit, respectively, followed by MA72 M131; MA100 P1S6 and MA100 M131 that improved nodulation by more than 140%. The effect of these combinations was even better than phosphate application (Figure 1). Nitrogen application showed the least number of nodules, even less to control. This might be explained by the nitrate inhibition of nodulation [58,59,60].

Nodule dry weight was slightly enhanced in co-inoculated treatments over the control. However, the difference was not significant except for the combinations MA72 P1S6 and MA100 P1S6 (120%) in Merchouch and MA72 P1S6, MA72 M131, and MA72 P80 (150%) in Ain Sbit (Tables S1–S2 in Supplemented data).

Cross section of nodules from inoculated treatments showed pink to red color, indicating that they were effective in nitrogen fixation compared to the most of nodules of non-inoculated treatments that showed green color. This result indicates that indigenous rhizobial population was non-efficient and strains used for chickpea inoculation were more competitive, infective, and efficient.

**Figure 2:**
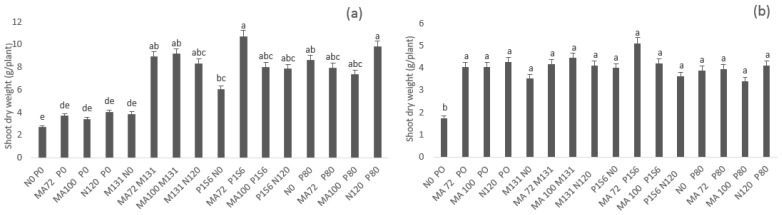
Effect of co-inoculation with *Mesorhizobium* spp. (MA72 or MA100) and *Bacillus* sp. (M131) or *E. aerogenes* (P1S6) and phosphate application combined to rhizobial inoculation on shoot dry weight of chickpea in Merchouch (**a**) and Ain Sbit (**b**) sites. Bars followed by the same letter are not significantly different at α = 0.05. (P80 indicate application of 80 kg P2O5/ha, N120 indicate application of 120 kg N /ha, N0 and P0 indicate no nitrogen and phosphate chemical fertilizers application).

Shoot dry weight was significantly (α = 0.05) enhanced for all inoculated treatments compared to control (2.68 g/plant in Merchouch and 1.75 g/plant in Ain Sbit) in both sites. However, this enhancement was related to different treatments and combinations. In Merchouch, the highest shoot dry weight was obtained by MA72 P1S6 strains combination (299%) followed by MA100 M131 (242%), and MA72 M131 (233%). The effect of these combinations was similar to the effect of mineral N and P fertilization in terms of shoot dry weight. In Ain Sbit, the highest shoot dry weight was obtained by MA72 P1S6 combination that increased the biomass with 192% compared to control (Figure 2).

**Figure 3:**
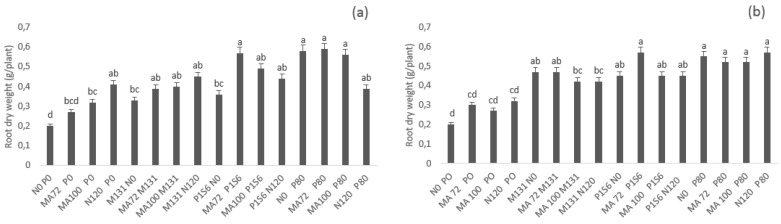
Effect of co-inoculation with *Mesorhizobium* spp. (MA72 or MA100) and *Bacillus* sp. (M131) or *E. aerogenes* (P1S6) and phosphate application combined to rhizobial inoculation on root dry weight of chickpea in Merchouch (**a**) and Ain Sbit (**b**) sites. Bars followed by the same letter are not significantly different at α = 0.05. (P80 indicate application of 80 kg P2O5/ha, N120 indicate application of 120 kg N /ha, N0 and P0 indicate no nitrogen and phosphate chemical fertilizers application).

In terms of root dry weight, there was no significant (α = 0.05) difference between different treatments in both sites apart from MA72 P1S6, MA72 P80, MA100 P80, and N0 P80 that resulted in approximately 185% root dry weight enhancement as compared to non-phosphate fertilized treatments in both sites. In Ain Sbit, the effect of these treatments was similar to the effect of N and P fertilization N120 P80 (Figure 3).

To estimate nitrogen fixation, we calculated the percent of nitrogen derived from air (% Ndfa). The estimated amount of fixed nitrogen is very rough because of the possibility for the influence of other soil microbes or inoculation on soil nutrient status, and thus on the intake of mineral forms of nitrogen [61,62]. Inoculation of chickpea with rhizobium strains MA72 or MA100 combined with M131 or P1S6 or P fertilizer resulted in a significantly higher % of nitrogen derived from air in both sites as compared to single inoculation with MA72 or MA 100 (Table 1). These observations suggest that dual inoculation of chickpea with used rhizobia and PSB enhanced significantly nitrogen fixation.

### 2.2. Effect of Co-Inoculation and Application of N and P Fertilization on Grain Yield, Straw Yield and Grain P, N, and Protein Content under Low Phosphate Conditions

**Figure 4:**
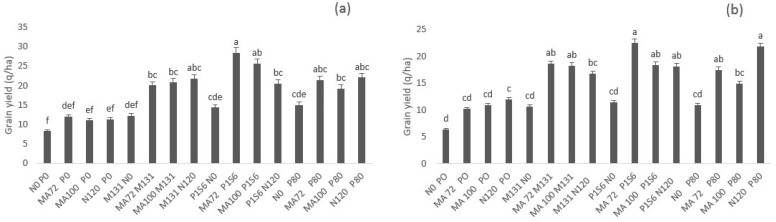
Effect of co-inoculation with *Mesorhizobium* spp. (MA72 or MA100) and *Bacillus* sp. (M131) or *E. aerogenes* (P1S6) and phosphate application combined to rhizobial inoculation on the grain yield of Chickpea in Merchouch (**a**) and Ain Sbit (**b**). Bars followed with the same letter are not significantly different at α = 0.05. (P80 indicate application of 80 kg P2O5/ha, N120 indicate application of 120 kg N /ha, N0 and P0 indicate no nitrogen and phosphate chemical fertilizers application).

At final harvest, grain yield in both sites was significantly enhanced (α = 0.05) by inoculation and /or fertilization over the control N0P0, except for MA72 P0, MA100 P0 and N120 P0 combinations for which the increase was not significant (α = 0.05); this could be due to the P deficit. This increase was enhanced with dual inoculation or phosphate fertilization. The highest yield was obtained by MA72 P1S6 that enhanced grain yield by approximately 250% (28.4 q/ha in Merchouch, 22.5 q/ha in Ain Sbit) compared to the control N0P0 (8.3 q/ha in Merchouch, 6.4 q/ha in Ain Sbit). The effect of this combination on yield was even better than P and N fertilization (22.2 q/ha in Merchouch, 21.8 q/ha in Ain Sbit). The effect of MA72 P1S6 on grain yield was followed by the effect of MA100 P1S6, MA100 M131, and MA72 M131. The effect of these combinations was statistically similar (α = 0.05) to the effect of single inoculation with rhizobia combined with P fertilization (MA100 P80, MA72 P80) and to the effect of combined fertilization (Figure 4).

Similar observations were obtained for the straw yield, the best yield was observed in MA72 P1S6 and MA100 P1S6 combinations, and their effect was higher than the effect of N and P fertilization (N120 P80). The effect of MA72 P1S6 and MA100 P1S6 on straw yield was followed by the effect of MA72 M131 and MA100 M131, and the difference between these combinations was not significant (α = 0.05). However, it was highly significant over the control N0P0 (Tables S1–S2).

These results showed that the effect of dual inoculation with rhizobia and PGPRs is similar to the effect of fertilization with N and P together, and resulted in a higher yield as compared to single inoculation with rhizobia or single application of fertilizers.

**Figure 5:**
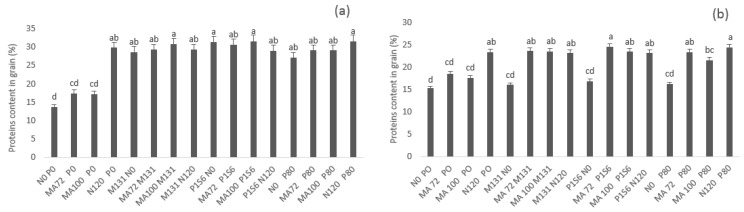
Effect of co-inoculation with *Mesorhizobium* spp. (MA72 or MA100) and *Bacillus* sp. (M131) or *E. aerogenes* (P1S6) and phosphate application combined to rhizobial inoculation on proteins content in grain of Chickpea in Merchouch (**a**) and Ain Sbit (**b**). Bars followed with the same letter are not significantly different at α = 0.05. (P80 indicate application of 80 kg P2O5/ha, N120 indicate application of 120 kg N /ha, N0, and P0 indicate no nitrogen and phosphate chemical fertilizers application).

In terms of N, P, and protein content in grains, all the treatments showed a significant improvement over the control N0 P0 except for single inoculation with rhizobia that did not show a significant enhancement compared to control because of the limited amount of P in soils (Figure 5; Tables S1–S2)

## 3. Discussion

In the present study, we demonstrated the effect of chickpea inoculation with *Mesorhizobium* sp. MA72 and *Mesorhizobium ciceri* MA100 combined to PGPRs (*Bacillus* sp. (M131) and *E. aerogenes* (P1S6)) or Phosphate fertilization under P-deficient conditions. Obtained results revealed that co-inoculation results in an increase of nodulation (up to 271% in Merchouch and 305% in Ain Sbit), shoot dry weight (up to 299% in Merchouch and 192% in Ain Sbit), root dry weight (up to 185% in both sites), grain yield (up to 242% in Merchouch and 251.5% in Ain Sbit), % Ndfa and P, N and proteins content in grains as compared to single inoculation, single N and P fertilization and control. Suggesting that indigenous rhizobia are inefficient or inappropriate for chickpea nodulation in such soil conditions. The effect of MA72 P1S6 was always better than the effect of the other rhizobium-PGPR combinations. However, its effect was not statistically significantly different from the effect of the other combinations and mineral fertilization. This enhancement was equivalent or even higher to combined N and P fertilization probably by providing a more nutrient balance to plant, indicating that used rhizobia were more effective nitrogen fixers as well as M131 and P1S6 were more effective phosphate solubilizers and plant growth promoters than native bacterial population. Obtained results of Merchouch were slightly higher than in Ain Sbit, and that can be explained by the clay nature of the soil in Merchouch, which retains more water compared to Ain Sbit.

Used PGPRs are characterized by their ability to solubilize phosphate. However, this bacterial P-solubilization is generally due to excretion of organic acids such lactic acid, citric acid, oxalic acid, etc. which can limit the development of rhizobia that prefer neutral or alkaline conditions during nodulation [63]. In addition, analysis of soil pH in Merchouch indicated that it is an acidic soil, and, therefore, we used acid tolerant rhizobia (MA72 and MA100; [9]) for inoculation to alleviate the effect of these produced acids during P solubilization.

Single rhizobial inoculation supplemented with P- fertilization at a level of 80 kg P_2_O_5_/ha (35 kg P/ha), resulted in higher nodule number and dry weight as compared to single rhizobial inoculation. These observations were similar to findings of Ben Romdhane et al. [64,65] reporting nodulation enhancement by phosphate application. The low number of nodules in the absence of phosphate fertilization might be explained by the limited available P in the studied soil, since phosphorus is included in ATP synthesis which is essential for nodules formation and nitrogen fixation [66]. Many researchers reported the importance of P supplements with rhizobium inoculation for soil fertility because of its potential for good nitrogen fixation by increasing nodulation in legumes [41,67]. Moreover, Jebara et al. [68] reported that up to 20–25% of total plant P is required for nodule fraction. Furthermore, enhancement of chickpea nodulation as a result of phosphate application with rhizobial inoculation was reported by Wolde-meskel et al. [5]. Similarly, Sumit et al. [69] reported that inoculation of cowpea with a nitrogen fixing bacterium (cyanobacterial inoculant) supplemented with P fertilization enhanced rooting and symbiotic traits related to nodulation, nitrogen fixation, and nutrient uptake in cowpea crop. Likewise, Kyei-Boahen et al. [70] reported that rhizobial inoculation with mineral P fertilization improved cowpea-*Bradyrhizobium* symbiosis efficiency compared to either inoculant or P applied alone.

Through their ability to solubilize phosphate, P1S6 and M131 isolates mitigate the effect of phosphate fertilization to enhance nodulation in chickpea; their effect was similar or even higher than the effect of P-application. Enhancement of nodule number and dry weight due to combined inoculation might be explained by the expansion in root length and mass resulting in more active sites for nodulation by the rhizobial strains as reported by Korir et al. [48] and Dumsane et al. [51]. These results were similar to findings of Rudresh et al. [71] and Elkoca et al. [72] reporting that co-inoculation of chickpea with rhizobia and PSB such as *Bacillus subtilis* and *Bacillus megaterium* resulted in a higher nodulation and seed yield. Similarly, Zafar et al. [73] reported that co-inoculation with rhizobia and PGPRs enhance top, root, and nodulation of *Phaseolus vulgaris*.

These findings suggest that used PGPRs (M131 and P1S6) act synergistically with rhizobial strains (MA72 and MA100) in promoting nodules’ initiation or development. Promotion of shoot and root growth might be explained by the improvement of mineral nutrition due to co-inoculation as it was reported by Burdman et al. [74] and Cakmakçi et al. [75]. In addition, the increase in shoot dry weight of chickpea may be attributed to increased root proliferation induced by used rhizobia or PSB or both, promoting nutrient and water uptake by roots of chickpea [76].

The present results are supported by findings of Valverde et al. [77] reporting that co-inoculation of chickpea with *P. jensenii* PS06 and *M. ciceri* C2/2 enhanced nodulation and seed yield. Furthermore, Wani and Khan [78] reported that chickpea inoculation with *bacillus* sp. improved significantly growth, nodulation, chlorophyll, seed yield, and grain proteins. Moreover, Verma et al. [79] showed that a maximum increase in nodule number and dry weight, shoot, and root biomass was observed while co-inoculating chickpea with *Rhizobium* sp. BHURC01 and *Pseudomonas fluorescence.* Furthermore, Dumsane et al. [51] reported that under a P-deficient soil, inoculation of white clover with *Rhizobium* in combination with two PGPRs, *Bacillus aryabhattai* strain Sb and *Azotobacter vinelandii* strain G31, enhanced significantly dry weight, nodule number, and nitrogenase activity as compared to simple rhizobium inoculation.

In terms of grain and straw yield, the effect of dual inoculation was similar to the effect of combined application of N and P fertilizers. This can be resulted from the enhanced P nutrition, the production of phytohormones and interactions between several biochemical factors and plant nutrient status of the soil as it was stated by Vurukonda et al. [80]. Moreover, all the co-inoculated treatments allowed a significant enhancement of N, P and protein content in grains as compared to control. This might be explained by the enhanced nutrient uptake resulting from enhanced root system, improved nitrogen fixation by rhizobia and phosphate solubilization by PSB.

These observations were in line with several reports on legumes co-inoculation. Findings of Wolde-meskel et al. [5] in Ethiopian soils showed that P fertilization of chickpea combined with rhizobia inoculation resulted in 38% increase as compared to non-inoculated and non-fertilized treatments. Similarly, results of Verma et al. [79] highlighted the positive effect of chickpea co-inoculation with *Mesorhizobium* sp. and *P. aeruginosa* that resulted in a 32% increase in grain yield and 41% in straw yield as compared to uninoculated control. Analogous observations were reported on the effect of co-inoculation of other legumes such as peagen pea [81], common bean [48,65], pea [82,83] white clover [51], lentil [84], and faba been [50].

## 4. Materials and Methods

### 4.1. Bacterial Strains Used in This Study

The strains *Mesorhizobium* sp. MA72 and *Mesorhizobium ciceri* MA100 used in this study were isolated from chickpea nodules collected from chickpea fields in Morocco and characterized in our previous study [9]. They were tolerant to acidic pH 5.5–6.0 and other abiotic stresses (cold temperature of 4 °C, high temperature 36–40 °C, 513–684 mM of NaCl) and showed better symbiotic performance with chickpea [9].

The pure cultures of PGPR M131 (*Bacillus* sp.) isolated from the inside chickpea nodules and P1S6 (*Enterobacter aerogenes*) isolated from the rhizosphere of lentil, were provided by Microbial and Molecular Biology Unit—Center of Plant and Microbial Biotechnology—Faculty of Science, Mohammed V University, Rabat, Morocco. These two PGPRs showed high ability to solubilize inorganic phosphate and to produce siderophores in vitro (data not shown).

These rhizobia (MA72 and MA100) and phosphate solubilizing bacteria (PSB; P1S6 and M131) were grown on YEM solid medium with 1.8% of agar [85] at 28 °C.

### 4.2. Assessment of Co-Inoculation of Chickpea with Mesorhizobium spp. and Bacillus sp. or E. aerogenes

#### 4.2.1. Plant Material and Experimental Sites

A Moroccan chickpea variety Zahor (F84-182C) was used for co-inoculation. This variety has large seed size, resistant to Ascochyta blight and suitable for winter planting.

The experiment was laid-out in two sites in Morocco: Merchouch (33°36.656′ N 006°43.216′ W) and Ain Sbit (33°32.384′ N 006°30.938′ W), both are in Rabat-Sale-Zemour-Zair (RSZZ) region characterized with a semi-arid climate. The average rainfall is 449 mm and 464 mm in Merchouch and Ain Sbit, respectively. The mean temperature is approximatively 17 °C in both sites. Soil samples (0–30 cm depth) from the study sites were collected to characterize pH, electrical conductivity, organic matter, nitrogen, phosphate (P), and K content. The field chosen sites had no previous history of chickpea cultivation or inoculation of rhizobia.

#### 4.2.2. Inoculum Production

*Pre-inoculum preparation:* Liquid inoculum was prepared by inoculating MA72, MA100, P1S6 and M131 into 200 mL of liquid YEM broth [85]. These suspensions were incubated at 28 °C for 72 h in a rotary shaker (at 200 rpm) to reach a bacterial culture of 10^9^ cfu/mL.

*Peat inoculation:* Peat was used as inoculum carrier. Peat was dried at 70 °C for 48 h, crushed to a fine powder (0.2 mm), neutralized using CaCO_3_ (1 g of CaCO_3_ per 100 g of peat) and sterilized. For single inoculum, 375 mL of each bacterial culture (10^9^ cfu/mL) was mixed with 250 g of sterilized peat powder. For PGPR-rhizobium inoculum, 187 mL of M131 or P1S6 culture (10^9^ cfu/mL) and 187 mL of MA72 or MA100 culture (10^9^ cfu/mL) mixed with 250 g of sterilized peat powder. Final peat-based inoculant contained 1.5 × 10^9^ cfu/g of peat.

#### 4.2.3. Seeds Inoculation

To stick the inoculant to seeds, adhesive was prepared one day before inoculation. 30 g of Arabic gum was dissolved in 1000 mL of near boiling distilled water with steering until the gum is dispersed. pH was adjusted to pH 7 using 1N NaOH.

To inoculate seeds, they were first mixed with the prepared adhesive (6 mL of adhesive/100 g of seeds). Then, prepared inoculated peat was immediately added to seeds coated with adhesive. Each 250 g of prepared inoculated peat was used to inoculate 1.25 kg of chickpea seeds, resulting in approximatively 10^7^ to 10^8^ cfu/seed.

The number of bacterial cells per inoculum and per seeds used for chickpea inoculation is based on previous research described by Remans et al. [65], Dobbelaere et al. [86] and Verma et al. [79].

#### 4.2.4. Field Experiment Design

Field experiments of chickpea co-inoculation with rhizobia (MA72 and MA100) and phosphate solubilizing bacteria (P1S6 and M131) were laid out at two sites, Merchouch and Ain Sbit, during 2017–2018.

Soils of experimental sites were non-saline and characterized with neutral pH in Ain Sbit and acid pH in Merchouch, moderate level of organic matter and nitrogen, low level of available P and high level of K [87]. The soils were classified as vertisol in Ain Sbit and fersiallitic in Merchouch [88]. Properties of the studied soils are presented in Table 2. The choice of these two types of soil was based on their representativeness area in chickpea growing regions in Morocco (Rabat-Sale-Kenitra region).

The experiments were carried-out in a randomized complete block design (RCBD) with four replications for each treatment. Sixteen treatments were applied in each site:

- N0 P0: Uninoculated and unfertilized treatment (control)
- MA72 P0: Seeds inoculated with MA72
- MA100 P0: Seeds inoculated with MA100
- N120 P0: Application of Nitrogen at a rate of 120 kg N/ ha, no inoculation was applied in this treatment
- M131 N0: Seeds inoculated with M131
- MA72 M131: Seeds inoculated with MA72 + M131
- MA100 M131: Seeds inoculated with MA100 + M131
- M131 N120: Seeds inoculated with M131 + Nitrogen application at a rate of 120 kg N/ ha
- P1S6 N0: Seeds inoculated with P1S6
- MA72 P1S6: Seeds inoculated with MA72 + P1S6
- MA100 P1S6: Seeds inoculated with MA100 + P1S6
- P1S6 N120: Seeds inoculated with P1S6 + Nitrogen application at a rate of 120 kg N/ ha
- N0 P80: Application of phosphate at a rate of 35 kg P/ha, no inoculation was applied in this treatment
- MA72 P80: Seeds inoculated with MA72 + phosphate application at a rate of 35 kg P/ha
- MA100 P80: Seeds inoculated with MA100 + phosphate application at a rate of 35 kg P/ha
- N120 P80: Application of 120 kg N/ ha and of 35 kg P/ha, no inoculation was applied in this treatment

Dimensions of each elementary plot were 4 m × 2 m each, with 1 m paths separating the plots. Blocks were spaced at 2 m and rows were spaced at 30 cm. Seeds were sown at a depth of 5–7 cm and spaced at 10 cm within rows. Uninoculated plots were sown before inoculated ones to avoid contamination [89].

All plots received potash at the rate of 80 kg/ha as 167 kg/ha of K_2_SO_4_. Nitrogen was applied at the rate of 120 kg N/ha as 260 kg/ha of urea. Phosphate was applied at the rate of 35 kg P/ha (80 kg P_2_O_5_/ha) as 177.7 kg/ha of Tri-Super-phosphate (TSP). All fertilizers were applied at the time of planting except for nitrogen application split as 60 kg N/ha was applied at pre-sowing and 60 kg N/ha at 50% flowering.

### 4.3. Data Collection

#### 4.3.1. Nodules Collection and Biomass Measurement

Four plants were randomly selected at the 50% flowering from each elementary plot (replication) and uprooted with soil intact in roots in order not to lose nodules. Soil from the roots was carefully washed out to ensure that nodules and roots were intact [89].

Roots, nodules, and shoots were separated, dried in oven at 75 °C for 72 h [2]. Number and dry weight of nodules per plant and dry weight of shoot and root were determined on four plants for each replication of the treatments.

#### 4.3.2. Grain Yield and Total Dry Matter

Plants from 1 m^2^ area of unsampled rows from each plot were harvested at maturity. Samples were dried at ambient temperature for 15 days. Total dry matter was determined before threshing. Seeds were weighed and grain and straw yield per hectare were calculated.

#### 4.3.3. Nitrogen, Phosphate, and Protein Content in Grain

The seeds were grounded and a sample of ground seeds (0.2 g) from each plot were used to determine N and P content. For the analysis of total N, samples were grounded and digested in 10 mL mixture of 9:1 ratio of H_2_SO_4_:HClO_4_ (Nessler’s reagent method) [79,90]. Grain protein content was determined according to the formula: Nitrogen (%) × 6.25 [79]. For P content, samples were digested in 10 mL of 4:1 ratio of HNO_3_:HClO_4_ [91].

#### 4.3.4. Nitrogen Derived from the Atmosphere

The percent of nitrogen derived from the atmosphere (% Ndfa) was calculated according to the following formula:

% Ndfa = 100 (TPNinc − TPNcont)/TPNinc(1)

where TPNinc is the total N content in inoculated plants, and TPNcont is the total N content in non-inoculated plants [51,92].

### 4.4. Statistical Analysis

Data were collected in four replicates (4 plants per replication in 4 plots) and were analyzed using SAS Statistical Package Version 9.3. A plot replication was considered as the experimental unit. Analysis of variance at 95% using GLM (Generalized linear models) was performed to determine the effect of inoculation and/or fertilization. Duncan Multiple Range Test (DMRT) was used to compare treatment means at α = 0.05. Significant differences at α = 0.05 are indicated by different letters.

## 5. Conclusions

In the present investigation, we assessed the effect of combined inoculation of chickpea with *Mesorhizobium* spp. and *Bacillus* sp. or *E. aerogenes* with or without mineral N and P application in phosphate-deficient soils. Obtained results revealed that rhizobial inoculation combined with P fertilization resulted in a higher nodulation, biomass, and yield of chickpea as compared to single inoculation with rhizobium or single use of N or P. However, the high cost of mineral fertilizers and their negative effect on the environment emphasizes the importance of development of a cheap eco-friendly alternative using phosphate-solubilizing bacteria (PSB) as a viable continuous source of phosphate.

Hence, the present study highlighted the synergetic effect of used rhizobia and PSB (*Bacillus* sp. or *E. aerogenes)*, which resulted in a higher nodulation, biomass production, grain, and straw yield, uptake of P and N, and protein content in grains as compared to single inoculation of chickpea in P-deficient soils. The effect of these combinations was equivalent to the effect of inorganic fertilizers N and P. The best enhancement in this field experiment was obtained by MA72 P1S6 combination in Merchouch and Ain Sbit sites, which suggest using MA72 (*Mesorhizobium* sp.) and P1S6 (*E. aerogenes*) in formulation for bioinoculum product in chickpea cultivation as an economic substitute for N and P fertilizers for a sustainable agriculture. More field trials are needed to confirm the obtained results on a large scale.

## Figures and Tables

**Table 1:** Effect of co-inoculation with rhizobia and PSB or phosphate fertilization on the percent of N derived from air.

Treatment	% Ndfa in Ain Sbit	% Ndfa in Merchouch
MA72	22.26 ± 8.1 bc	16 ± 4.1 b
MA72 M131	52.18 ± 3.2 a	48 ± 7.6 a
MA72 P1S6	57.6 ± 2.8 a	50.94 ± 5.3 a
MA72 P80	51.9 ± 6.3 a	48.5 ± 3.7 a
MA100	16.78 ± 5.6 c	16.12 ± 1.3 b
MA100 M131	52.21 ± 2.7 a	51.13 ± 2.1 a
MA100 P1S6	51.41 ± 5.1 a	52.38 ± 6.6 a
MA100 P80	58.39 ± 2.7 a	52.68 ± 6.4 a

Values are mean of four replicates, mean values (mean ± SD). Means followed by the same letter within a column are not significantly different at α = 0.05.; Ndfa: Nitrogen derived from air.

**Table 2:** Soil properties of experimental fields.

Site	Mean Rainfall (mm) ^a^	Mean Temperature (°C) ^a^	Climate	Soil Properties						
				pH (HCl)	pH (H_2_O)	P (ppm) ^b^	K (ppm) ^b^	Total N (%)	Organic Matter (%)	EC (ds/m) ^c^
Merchouch	449	17.1	Semi-arid	5.7	5.0	6.72	189.27	0.4	2.5	1.6
Ain Sbit	464	17.3	Semi-arid	7.9	6.9	4.49	364	0.5	2.9	2.0

^a^ Average data of 10 years; ^b^ ppm = mg/kg of soil; ^c^ Saline soil: EC > 4 ds/m; Normal soil: EC < 4 ds/m.

## Data Availability

This study did not report any data.

## Supplementary Materials

The following are available online at https://www.mdpi.com/2223-7747/10/3/571/s1, Table S1: Effect of co-inoculation and/or mineral fertilization on straw yield, Nitrogen and Phosphate content in grain of chickpea in Merchouch, Table S2: Effect of co-inoculation and/or mineral fertilization on straw yield, Nitrogen and Phosphate content in grain of chickpea in Ain Sbit.
