# Supplementary material for: Co-Inoculation of Mesorhizobium ciceri with Either Bacillus sp. or Enterobacter aerogenes on Chickpea Improves Growth and Productivity in Phosphate-Deficient Soils in Dry Areas of a Mediterranean Region
Source: Plants (Basel). 2021 Mar 17;10(3):571. doi: 10.3390/plants10030571 (PMC8002673; doi:10.3390/plants10030571)
Supplement: Supplementary file 1 [file plants-10-00571-s001.pdf]

Supplementary Data

Table S1. Effect of co-inoculation and/or mineral fertilization on straw yield, Nitrogen and Phosphate content in grain of chickpea in Merchouch.

Treatment	Nodules dry weight (g/plant)	Straw yield (q/ha)	P content in grain (%)	N content in grain (%)
N0 P0	0.15 ± 0.03 b	51.1 ± 3.2 de	0.18 ± 0.02 b	2.20 ± 0.12 c
MA72 P0	0.15± 0.02 b	87.8 ± 2.7 ab	0.21 ± 0.03 b	2.80 ± 0.04 bc
MA100 P0	0.15± 0.03 b	89.5 ± 2.1 ab	0.23 ± 0.02 b	2.75 ± 0.06 bc
N120 P0	0.12± 0.02 b	54.3 ± 0.9 de	0.20 ± 0.02 b	4.77 ± 0.13 ab
M131 N0	0.17± 0.01 b	89.2 ± 2.2 ab	0.40 ± 0.01a	4.59 ± 0.08 ab
MA72 M131	0.24± 0.04 ab	79.8 ± 3.1 bc	0.38 ± 0.02 a	4.69 ± 0.02 ab
MA100 M131	0.23 ± 0.02 ab	87.8 ± 3.1 ab	0.38 ± 0.02 a	4.94 ± 0.16 a
M131 N120	0.12± 0.02 b	81.9 ± 3.6 ab	0.37 ± 0.02 a	4.69 ± 0.14 ab
P1S6 N0	0.18± 0.05 b	59.0 ± 0.7 de	0.36 ± 0.06 a	5.01 ± 0.09 a
MA72 P1S6	0.33± 0.02 a	105.2 ± 2.1 a	0.38 ± 0.02 a	4.90 ± 0.06 ab
MA100 P1S6	0.32± 0.01 a	104.8 ± 3.6 a	0.41 ± 0.01 a	5.06 ± 0.12 a
P1S6 N120	0.13± 0.03 b	106.0 ± 3.9 a	0.40 ± 0.02 a	4.64 ± 0.13 ab
N0 P80	0.2± 0.06 ab	76.3 ± 3.6 bc	0.44 ± 0.02 a	4.35 ± 0.16 ab
MA72 P80	0.24± 0.03 ab	80.5 ± 2.1 bc	0.38 ± 0.06 a	4.65 ± 0.12 ab
MA100 P80	0.24± 0.02 ab	76.1 ± 3.8 bc	0.43 ± 0.02 a	4.65 ± 0.14 ab
N120 P80	0.15± 0.02 b	72.6 ± 3.1 bc	0.39 ± 0.03 a	5.06 ± 0.18 a

Values are mean of four replicates. Mean values (mean ± SD). Means followed by the same letter within a column are not significantly different at $\alpha = 0.05$.

Table S2. Effect of co-inoculation and/or mineral fertilization on straw yield, Nitrogen and Phosphate content in grain of chickpea in Ain Sbit.

Treatment	Nodules dry weight (g/plant)	Straw yield (q/ha)	P content in grain (%)	N content in grain (%)
N0 P0	0.12 ± 0.01 c	20.3 ± 3.2 d	0.15 ± 0.02 d	2.44 ± 0.12 d
MA72 P0	0.16 ± 0.01 bc	38.4 ± 1.2 ab	0.18 ± 0.01 d	2.95 ± 0.17 dc
MA100 P0	0.15 ± 0.02 bc	33.4 ± 2.2 bc	0.24 ± 0.01 cd	2.8 ± 0.21 dc
N120 P0	0.12 ± 0.01 c	33.0 ± 2.3 bc	0.26 ± 0.01 cd	3.73 ± 0.24 ab
M131 N0	0.15 ± 0.01 bc	37.0 ± 1.7 ab	0.37 ± 0.03 abc	2.59 ± 0.17 dc
MA72 M131	0.21 ± 0.01 ab	49.8 ± 3.5 a	0.47 ± 0.01 a	3.77 ± 0.15 ab
MA100 M131	0.12 ± 0.02 c	43.8 ± 2.2 ab	0.42 ± 0.03 ab	3.76 ± 0.13 ab
M131 N120	0.16 ± 0.01 bc	51.4 ± 2.6 a	0.42 ± 0.02 ab	3.70 ± 0.21 ab
P1S6 N0	0.12 ± 0.02 c	35.4 ± 2.8 bc	0.45 ± 0.02 a	2.72 ± 0.11 dc
MA72 P1S6	0.3 ± 0.01 a	56.2 ± 3.2 a	0.46 ± 0.01 a	3.92 ± 0.13 a
MA100 P1S6	0.2 ± 0.01 ab	51.3 ± 2.8 a	0.42± 0.02 ab	3.75 ± 0.18 ab
P1S6 N120	0.12 ± 0.01 c	23.3 ± 3.2 d	0.46 ± 0.01 a	3.70 ± 0.27 ab
N0 P80	0.16 ± 0.01 bc	20.7 ± 2.2 d	0.45 ± 0.03 a	2.62 ± 0.16 dc
MA72 P80	0.28 ± 0.01 a	31.8 ± 1.9 bc	0.44 ± 0.02 ab	3.74 ± 0.21 ab
MA100 P80	0.21 ± 0.01 ab	37.4 ± 1.2 ab	0.43 ± 0.04 ab	3.6 ± 0.19 bc
N120 P80	0.15 ± 0.02 bc	38.3 ± 2.5 ab	0.37 ± 0.02 abc	3.90 ± 0.11 a

Values are mean of four replicates. Mean values (mean ± SD). Means followed by the same letter within a column are not significantly different at $\alpha = 0.05$.
